# New Year, New JBO Editor-in-Chief

**DOI:** 10.1117/1.JBO.30.1.010101

**Published:** 2025-01-30

**Authors:** Muyinatu A. Lediju Bell

## Abstract

JBO Editor in Chief Muyinatu Bell highlights gratitude and forthcoming initiatives for the new year.

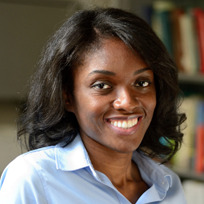



Dear JBO Community,

I am truly honored and excited for the opportunity to lead our journal as the new Editor-in-Chief (EIC). This journal is special to me because it published some of my early, pioneering work on photoacoustic imaging for surgical guidance, which then became the basis for many projects within my lab — the Photoacoustic and Ultrasonic Systems Engineering (PULSE) Lab — at Johns Hopkins University.

I am grateful to former EIC Brian Pogue for passing the torch to me, after a history of multiple successful EICs, including Prof. Lihong Wang and the current NIH NIBIB Director Bruce Tromberg. Each of these former EICs, and truly amazing individuals, are incredibly kind and appreciated for taking the time to share their congratulations, support, thoughts, and/or insightful perspectives at multiple points during my transition to EIC. I am also grateful to my active and engaged editorial board members for being the outstanding scientists and visionary leaders you are, who help to shape the direction and stature of our journal. And, of course, a million thanks over to our fantastic SPIE support staff for helping to make my transition to EIC as smooth as possible for me, as well as for supporting my authorship and editorial efforts prior to becoming EIC, and for all of the support already provided and additional support to come in my role as EIC.

Looking forward, I hope to see JBO remain at the forefront of innovations in biomedical optics. Considering how this community, and JBO in particular, welcomed me with open arms when I was a newcomer to the field, I will seek to do the same for our early-career members. I will support existing initiatives, such as the election of JBO Hot Topics speakers for Photonics West and collaborate with the Biophotonics and Imaging Summer School to publish related tutorials, as well as introduce new initiatives with the goal of building and strengthening ties to our SPIE conferences.

Five new initiatives that I intend to roll out over the next few years include the following:

1. Recurring topical special issues tied to at least three specific conference tracks at SPIE Photonics West. Conference chairs and committee members who want to be a part of this initiative are invited to contact me with proposals and expressions of interest in this opportunity.
2. Annual emerging investigator special issues, dedicated to the work from the laboratories of newly appointed assistant professors.
3. New options to link and/or advertise published SPIE Photonics West presentations and submitted posters with accepted JBO papers. For example, one option is to include presentation videos published in the SPIE Digital Library as video abstracts of a related JBO publication.
4. Clarification of the unique scope of JBO relative to related SPIE sibling journals (e.g., *Biophotonics Discovery*, *Neurophotonics*) for authors whose work may intersect multiple journals. As a start, my vision is that JBO will continue to focus on new innovations in biomedical optics, including innovations in technology, algorithms, or applications that have never been demonstrated before.
5. Collaborative co-authorship opportunities for presenters participating in JBO-sponsored webinars who are not already contributing to a JBO special issue, possibly to summarize the content of presented webinar material in a written, archivable format.

All of these and many other plans in store will only be possible with the support of a fantastic community of authors and reviewers, i.e., you! There are multiple reasons why your support is important to me, one of which being that JBO is a society journal. Hence, support from all members of our biomedical optics SPIE society is essential for this society journal to thrive. I am open to new ideas from our community and invite you to partner with me on this exciting venture.

Sincerely,

Muyinatu A. Lediju Bell

Editor-in-Chief

